# Tuberculosis in pediatric patients treated with anti-TNFα drugs: a cohort study

**DOI:** 10.1186/s12969-015-0054-4

**Published:** 2015-12-03

**Authors:** Joan Calzada-Hernández, Jordi Anton-López, Rosa Bou-Torrent, Estíbaliz Iglesias-Jiménez, Sílvia Ricart-Campos, Javier Martín de Carpi, Carmen García de Vicuña Muñoz de la Nava, Vicenç Torrente-Segarra, Judith Sánchez-Manubens, Clara Giménez-Roca, Librada Rozas-Quesada, Maria Teresa Juncosa-Morros, Clàudia Fortuny, Antoni Noguera-Julian

**Affiliations:** Pediatric Rheumatology Unit, Pediatrics Department, Hospital Sant Joan de Déu, Universitat de Barcelona, Esplugues, Spain; Pediatric Gastroenterology Department, Hospital Sant Joan de Déu, Universitat de Barcelona, Esplugues, Spain; Ophthalmology Department, Hospital Sant Joan de Déu, Universitat de Barcelona, Esplugues, Spain; Unit for Control of Infectious Diseases, Hospital Sant Joan de Déu, Universitat de Barcelona, Esplugues, Spain; Microbiology Department, Hospital Sant Joan de Déu, Universitat de Barcelona, Esplugues, Spain; Pediatric Infectious Diseases Unit, Pediatrics Department, Hospital Sant Joan de Déu, Universitat de Barcelona, Passeig Sant Joan de Déu 2, 08950 Esplugues de Llobregat, Spain

**Keywords:** Inflammatory bowel disease, Interferon-gamma release assays, Juvenile idiopathic arthritis, Tuberculosis, Anti-tumor necrosis factor-alpha drugs

## Abstract

**Background:**

Adult patients receiving anti-TNFα drugs are at increased risk of tuberculosis (TB), but studies in pediatric populations are limited, and the best strategy for latent tuberculosis infection (LTBI) screening in this population remains controversial. We describe the prevalence of LTBI prior to anti-TNFα therapy and the long-term follow-up after biological treatment initiation in a cohort of children and adolescents.

**Methods:**

Cohort observational study in children and adolescents receiving anti-TNFα agents in a tertiary-care pediatric hospital. LTBI was ruled out prior to the implementation of anti-TNFα drugs by tuberculin skin test (TST), and, from March 2012 on, QuantiFERON Gold-In Tube^®^ test (QTF-G). During anti-TNFα treatment, patients were evaluated every 6 months for TB with history and physical examination. TST/QTF-G were not repeated unless signs or symptoms consistent with TB arose or there was proven TB contact.

**Results:**

The final cohort consisted of 221 patients (56.1 % female; 261 treatments), of whom 51.7 %/30.0 %/17.3 % were treated with etanercept/adalimumab/infliximab, respectively, for a variety of rheumatic diseases (75.6 %), inflammatory bowel disease (20.8 %), and inflammatory eye diseases (3.6 %). The median (IQR) age at diagnosis of the primary condition was 6.8 years (2.7–11.0) and the duration of the disease before implementing the anti-TNFα agent was 1.8 years (0.6–4.2). LTBI was diagnosed in 3 adolescent girls (prevalence rate: 1.4 %; 95 % CI: 0.4–4.2) affected with juvenile idiopathic arthritis: TST tested positive in only 1, while QTF-G was positive in all cases (including 2 patients already on etanercept). They all received antiTB chemoprophylaxis and were later (re)treated with etanercept for 24–29 months, without incidences. No incident cases of TB disease were observed during the follow-up period under anti-TNFα treatment of 641 patients-year, with a median (IQR) time per patient of 2.3 years (1.4–4.3).

**Conclusions:**

In our study, the prevalence of LTBI (1.4 %) was similar to that reported in population screening studies in Spain; no incident cases of TB disease were observed. In low-burden TB settings, initial screening for TB in children prior to anti-TNFα treatment should include both TST and an IGRA test, but systematic repetition of LTBI immunodiagnostic tests seems unnecessary in the absence of symptoms or known TB contact.

## Background

Anti-tumour necrosis factor alpha (TNFα) agents represent a major advance in the treatment of several autoimmune and autoinflammatory diseases, both in children and in adults. TNFα plays a critical role in the host response to tuberculosis (TB), having an essential role in the formation of the granuloma. Exposure to anti-TNFα drugs has been associated with reactivation of latent TB infection (LTBI) in adulthood, even after discontinuation of the treatment [1–3]. Therefore, current guidelines recommend screening for LTBI before anti-TNFα treatment implementation [2]. In children and adolescents on anti-TNFα therapy, data are still scarce and questions about the best strategy for LTBI screening and the risk of TB remain unanswered [4].

Our aims were to assess the prevalence of LTBI in a cohort of children and adolescents expected to be treated with anti-TNFα agents and to describe the incidence of TB during follow-up while on anti-TNFα treatment.

## Methods

We conducted a retrospective observational study in a large cohort of patients aged <18 years treated with anti-TNFα agents for a variety of inflammatory diseases in a tertiary-care pediatric centre (Hospital Sant Joan de Déu, Barcelona) from December 2004 to January 2013, in Catalonia (Northeast Spain). The incidence rate of TB in Catalonia has decreased from 21.6 per 100,000 population in 2004 to 16.3 in 2012. Because anti-TNFα agents are hospital-prescribed drugs, patients were identified using centralized pharmacy records. Exclusion criteria were: 1. previous history of LTBI and/or TB; 2. previous exposure to anti-TNFα drugs; 3. follow-up time <3 months since anti-TNFα drug implementation; and 4. anti-TNFα treatment first prescribed elsewhere. Patients for whom complete data on LTBI screening could not be retrieved were not included in the analysis of LTBI prevalence.

The following demographic and clinical variables were collected: age, gender, primary disease, time from diagnosis until start of anti-TNFα drug, and type and duration of anti-TNFα treatment. All patients are routinely screened for TB infection before anti-TNFα drug implementation. Our local protocol includes clinical history focused on TB risk (immigration from or travel to a high burden country for TB, known TB contacts, and signs or symptoms consistent with TB), Bacillus Calmette-Guérin (BCG) vaccine status, physical examination, tuberculin skin test (TST), and chest x-ray (CXR). A positive TST result is defined as a reaction ≥5 mm [5]. BCG vaccine is not recommended in Spain. As from March 2012, an IGRA (interferon-gamma release assays) test (Quantiferon-TB Gold-in Tube^®^ test, QTF-G; Cellestis, Carnegie, Australia) became available in clinical practice in our centre and was included in the screening protocol, together with TST. After initial assessment, patients are clinically re-evaluated every 3–6 months for TB infection, but CXR and TST/QTF-G are not routinely repeated in the absence of signs or symptoms consistent with TB or proven TB contact. In those patients with negative screening results before March 2012 who were still on anti-TNFα drugs later on, QTF-G was performed once during clinical follow-up. After exclusion of TB disease, any positive result is taken as evidence for LTBI and secondary chemoprophylaxis is recommended, as per national guidelines [5]. After 4 weeks of LTBI prophylaxis, anti-TNFα treatment is implemented if indicated. The local ethics committee approved the study. Categorical variables were described as percentages, and continuous variables as mean/standard deviation (SD) or median/interquartile ranges (IQR). Finally, we performed a systematic literature review to find original studies that evaluated anti-TNFα therapy in human subjects aged <18 years (English language, years 2000–2014). We reviewed all references found through PubMed using a combination of the search terms infliximab (INF), etanercept (ETN), adalimumab (ADA), and TB. We then checked the references of each study to find any missed studies.

## Results

From December 2004, a total of 261 anti-TNFα treatments were prescribed to 221 patients (124 females, 97 males). Thirty-six patients switched once and two patients switched twice to other anti-TNFα drugs, mainly due to insufficient clinical response. The median (IQR) age at diagnosis of the primary condition was 6.8 (2.7–11.0) years and the duration of the disease before implementing the anti-TNFα agent was 1.8 (0.6–4.2) years. Etanercept was the most commonly prescribed drug (51.7 %), followed by ADA (31.0 %) and INF (17.3 %). The total follow-up time while on anti-TNFα treatment was 614 patient-years; median time on anti-TNFα drug per patient was 2.3 (1.4–4.3) years (ETN 1.9 [1.8–3.7]; ADA 1.8 [1.2–2.6]; and INF 2.1 [1.4–3.3] years). Juvenile idiopathic arthritis (JIA) was the most frequent indication for anti-TNFα treatment (74.7 %, 195 treatments in 163 patients); other indications included inflammatory bowel disease (19.1 %, 50 treatments in 46 patients) and idiopathic uveitis (4.2 %, 11 treatments in 8 patients) (Table 1).

**Table 1:** Primary diseases leading to anti-TNFα treatment implementation; number (%) of patients in all cases

Primary disease	Patients (*n* = 221)	Treatments (*n* = 261)
Juvenile idiopathic arthritis	163 (73.6)	195 (74.7)
Polyarticular JIA, positive RF	6 (2.7)	7 (3.8)
Polyarticular JIA, negative RF	40 (18.1)	48 (18.4)
Oligoarticular JIA	70 (31.7)	88 (33.7)
Enthesitis-related arthritis	24 (10.9)	24 (9.2)
Psoriasic arthritis	11 (5.0)	13 (5.0)
Undifferentiated disease	12 (5.4)	15 (5.7)
Blau syndrome	1 (0.5)	2 (0.8)
Tumor necrosis factor associated periodic syndrome	1 (0.5)	1 (0.4)
PAPA syndrome	1 (0.5)	1 (0.4)
Chronic plantar fascitis	1 (0.5)	1 (0.4)
Inflammatory bowel disease		
Crohn’s disease	36 (16.3)	39 (14.9)
Ulcerative colitis	10 (4.5)	11 (4.2)
Inflammatory diseases of the eye		
Uveitis	7 (3.2)	10 (3.8)
*Pars planitis*	1 (0.5)	1 (0.4)

*JIA* juvenile idiopathic arthritis, *PAPA* pyogenic arthritis, pyoderma gangrenosum and acne, *RF* rheumatoid factor

Most of the patients were Spanish (197 children, 89.1 %). Among 16 patients from South America and 8 from Morocco, a BCG scar was documented in 13 and 4 children, respectively. Other than immigration from TB-endemic regions, no other risk factors for TB were identified. Retrospective chart review documented no abnormal findings on CXR for 212 patients (95.9 %) and negative TST results in 209 children (94.6 %); positive TST was observed only in patient 3 (Table 2). Baseline CXR and TST results were not available for 9 and 11 patients, respectively.

**Table 2:** Details of the 3 girls affected with juvenile idiopathic arthritis that were diagnosed with latent tuberculosis infection and received antituberculosis chemoprophylaxis

Pt	Age - gender – primary disease	TST (mm)	Treatment at QTF-G performance	QTF-G IFN-γ concentrations (TB antigens-nil)	LTBI chemoprophylaxis	Maximum ALT/AST (IU/L)	Time on anti-TNFα drugs (before/after LTBI chemoprophylaxis)
1	11y6m – female- polyarticular JIA, negative RF	0	ETN + MTX	0.55 IU/mL	H for 9 months	30/37	22 (1/24) months
2	10y7m – female - oligoarticular JIA	0	ETN	0.37 IU/mL	HR for 3 months	31/38	29 (14/25) months
3	8y5m – female - oligoarticular JIA	15	NSAIDs	4.78 IU/mL	HR for 3 months	25/24	12 (0/29) months

*ALT* alanine aminotransferase, *AST* aspartate aminotransferase, *H* isoniazid, *IFN-γ* interferon-gamma, *MTX* methotrexate, *NSAIDs* non-steroidal anti-inflammatory drugs, *Pt* patient, *R* rifampicin, *RF* rheumatoid factor

From March 2012, QTF-G was performed in 75 patients (33.9 %), with positive, negative, and indeterminate results in 3 (patients 1–3, Table 2), 66, and 6 children, respectively. Baseline TST was positive in just one of the 3 QTF-G positive patients, all of whom were BCG-unvaccinated. All indeterminate QTF-G results were due to low mitogen response. Baseline screening results in these six patients (median [range] age: 8.6 [7.0-12.9] years) had previously ruled out LTBI and they were all on anti-TNFα treatment, associated with methotrexate in 4 cases. QTF-G was repeated in 4 of the 6 children with previous indeterminate result and proved negative in 3 of them, while remaining indeterminate in one. None of these patients received chemoprophylaxis nor developed TB after a median (IQR) follow-up of 1.8 (1.6–1.9) years. The other two patients were transferred to adult care and were lost to follow-up.

Three Spanish girls affected with JIA were diagnosed with LTBI (prevalence rate: 1.4 %; 95 % confidence interval: 0.4–4.2 %; Table 2). Focused history disclosed no known risk of TB infection in any of the girls; they had not received BCG vaccine and no clinical or radiological evidence of TB was detected. In patient 3, both TST and QTF-G tested positive before anti-TNFα or any other immunosuppressive drug was implemented. In patients 1 and 2, baseline TST was negative but QTF-G tested marginally positive afterwards, when ETN had already been implemented. When they were diagnosed with LTBI, the anti-TNFα treatment was discontinued and anti-TB chemoprophylaxis started, either 9 months of isoniazid monotherapy or 3 months of isoniazid and rifampicin. All patients adhered properly to the LTBI chemoprophylaxis regimen and no tolerability problems were reported. Etanercept was resumed 1 month after anti-TB chemoprophylaxis implementation; clinical follow-up while on ETN for 24, 25, and 29 months in patients 1, 2, and 3, respectively, showed no evidence of TB reactivation . Overall, after a follow-up of 614 patient-years, no incident cases of TB disease were observed.

## Discussion

In adults, anti-TNFα agents increase the risk of LTBI reactivation [1–3]. The risk has been described as being higher with monoclonal antibodies (INF/ADA), as compared with ETN, and times to TB onset from anti-TNFα drug implementation are shorter with INF (median time: 5.5 months) than with ETN (13.4 months) or ADA (18.5 months) [1]. In our pediatric cohort, patients received both monoclonal antibodies and ETN for a median time of 2.3 years per patient, and no incident TB disease cases were observed. Both the use of those drugs with the higher risk and the long follow-up time in our cohort are reassuring.

A systematic review by Toussi et al. identified very few children on anti-TNFα drugs who developed TB [6], likely owing to a lower prevalence of LTBI in the pediatric age and to effective screening of LTBI. Table 3 summarizes all reported cases to date of mycobacterial disease in children and adolescents exposed to anti-TNFα agents [3, 4, 7–14]. All cases involved extrapulmonary TB, and miliary/meningeal TB and death were reported in 5 and 2 patients (out of 11 cases), respectively. Of note, disease caused by non-TB mycobacterial species has also been reported after anti-TNFα drug use [13], and also following *in utero* exposure to infliximab [14].

**Table 3:** Table summarizing cases published to date of mycobacterial disease in patients aged <18 years who were exposed to anti-TNFα agents

Pt	Ref	Primary disease	Anti-TNFα drug	Age/gender	Time on drug	Details on mycobacterial disease
Tuberculosis disease						
1	[3]	JIA	Etanercept	NR	NR	Extrapulmonary
2	[3]	JIA	Etanercept	NR	NR	Extrapulmonary
3	[7]	Polyarticular JIA	Infliximab + MTX	NR	2y	Asymptomatic miliary TB (MTB PCR positive in sputum) with good response to 9-month standard treatment
4	[8]	Systemic JIA	Etanercept → infliximab + (previous extensive immunosuppressive therapy)	9y / female	NR	Subcutaneous cyst on the left wrist, after extensive microbiological studies, only MTB PCR in cyst fluid was positive. The child died after fulminant undiagnosed opportunistic pulmonary infection, 1 month after anti-TB drugs were implemented
5	[9]	Systemic JIA	Etanercept (previous CT and MTX)	9y / female	5wk	Ankle arthritis with positive MTB culture and normal chest X-ray; good response to 12-month standard treatment
6	[10]	SAPHO syndrome	Adalimumab (previous CT)	17y / female	3y	Meningeal and miliary TB presenting with septic shock; several PCR and cultures tested positive for MTB. Good response to therapy, but for neurologic sequelae. Adalimumab stopped 4 weeks earlier, suggesting immune reconstitution inflammatory syndrome
7	[11]	Polyarticular JIA	Infliximab (previous MTX)	13y / male	3 m	TB pleuritis (cultures negative) with good response to anti-TB therapy
8	[12]	Ulcerative colitis	Infliximab (previous CT and azathioprine)	17y / male	8 m	Miliary TB; the patient developed isoniazid-related hepatitis, but showed good response later on to an isoniazid-free regimen
9	[12]	Crohn’s disease	Infliximab	13y / female	2y	Disseminated TB disease with good response to 9-month standard therapy
Nontuberculous mycobacterial disease						
10	[13]	Crohn’s disease	Infliximab → adalimumab (previous CT)	12y / female	9 m	Generalized lymphadenopathies due to culture-proven *Mycobacterium avium complex* infection; good response to ethambutol, clarithromycin and rifampin
11	[14]	Mother’s Crohn disease	Infliximab during gestation	3 m / male	36wk *in utero*	Bottle-fed. BCG vaccination at 3 m of age; disseminated BCGitis leading to death 6 weeks later

*BCG* bacillus Calmette-Guérin, *CT* corticosteroids, *MTB Mycobacterium tuberculosis*, *MTX* methotrexate, *NR* not reported, *PCR* polymerase chain reaction, *Pt* patient, *Ref* reference, *SAPHO* synovitis acne pustulosis hyperostosis and osteitis

To the best of our knowledge, this is the largest published cohort of patients aged <18 years treated with anti-TNFα agents in whom TB infection was investigated, both in terms of number of children and length of follow-up. The LTBI prevalence rate in our study (1.4 %) is similar to that reported in the healthy pediatric Spanish population (0.9 %) [15], and, more importantly, no incident cases of TB disease were observed. The retrospective design, the low TB incidence in Catalonia, and the fact that QTF-G was unavailable until March 2012 are limitations of our study.

Similar studies from Turkey and England have been published [16–19]. In the Turkish studies, the reported prevalence rate for LTBI ranged from 4.8 to 19.4 %, and this was probably overestimated because of false-positive results due to universal BCG vaccination in Turkey [16–18]. In the English study, both TST and QTF-G were performed by INF treatment in 23 children, and only one case of LTBI was diagnosed [19]. All LTBI cases in these studies received chemoprophylaxis before anti-TNFα drug implementation, and no cases of TB were reported, highlighting the importance of LTBI diagnosis and treatment in this group of patients. In the absence of specific data on the best chemoprophylaxis regimen for LTBI in these patients, using the same regimens used in healthy children seems reasonable [5].

The best way to screen for LTBI in these patients remains uncertain. Recent studies in adults with immune-mediated inflammatory diseases have demonstrated that IGRA tests: a) show a better association with classic risk factors for LTBI; b) are more specific than TST for the diagnosis of LTBI, especially among BCG-vaccinated patients, and therefore decrease the number of LTBI treatments needed; and c) are more sensitive than TST, probably because they are less affected by concomitant use of corticosteroids or other immunosuppressive drugs [20, 21]. Despite this, significant numbers of patients with discordant positive TST/negative IGRA results in the absence of BCG vaccination have also been observed [21]. The latest expert recommendations call for a dual testing strategy including both TST and an IGRA test in immunosuppressed patients, or even a dual IGRA strategy in regions using BCG, in order to maximise sensitivity [22].

For the pediatric age data are still very scarce. In a large longitudinal series of children with inflammatory bowel disease (72 patients, 165 QTF-G tests), indeterminate QTF-G results (10 %) were associated with corticosteroid use and several markers of disease activity, and 4 QTF-G conversions and 5 QTF-G reversions were identified during follow-up [23]. Similar rates of indeterminate IGRA results have been reported by other authors, and several cases of negative TST/positive IGRA and even negative/negative results in children with TB have been described [10, 19]. Interestingly, in our cohort, LTBI in patients 1 and 2 was only diagnosed with borderline positive QTF-G results when both girls were already on ETN. While this may represent false-positive results, it has also been reported that anti-TNFα therapy is associated with a higher rate of indeterminate IGRA results [23]. Given the lack of evidence, we are in agreement that both TST and at least one IGRA test should be used in immunosuppressed children, and any positive result should be taken as evidence of LTBI.

## Conclusions

No incident cases of TB disease were observed after a 614 patient-year follow-up in a cohort of children and adolescents receiving anti-TNFα drugs in a low-incidence setting. Three girls (1.4 %) were diagnosed with LTBI; they received anti-TB chemoprophylaxis and did well upon ETN resumption. Tuberculosis does not seem to be a prevalent problem in our population, but severe evolution in the cases reported to date makes LTBI screening mandatory prior to anti-TNFα drug implementation. Although further data are needed to better define the optimal LTBI testing strategy in these children in low-burden TB settings, initial screening should include both TST and an IGRA test. However, systematic repetition of LTBI immunodiagnostic tests seems unnecessary in the absence of symptoms or known TB contact.

## Abbreviations

ADA: Adalimumab
BCG: Bacillus Calmette-Guérin
CXR: Chest X-ray
ETN: Etanercept
IGRA: Interferon-gamma release assay
INF: Infliximab
JIA: Juvenile idiopathic arthritis
LTBI: Latent tuberculosis infection
QTF-G: Quantiferon-TB gold-in tube test
TB: Tuberculosis
TNFα: Tumor necrosis factor alpha
TST: Tuberculin skin test
